# Possible Health Benefits and Risks of DeepFake Videos: A Qualitative Study in Nursing Students

**DOI:** 10.3390/nursrep14040203

**Published:** 2024-10-03

**Authors:** Olga Navarro Martínez, David Fernández-García, Noemí Cuartero Monteagudo, Olga Forero-Rincón

**Affiliations:** 1 Nursing Education and Care Research Group (GRIECE), Nursing Department, Faculty of Nursing and Podiatry, Universitat de València, Menéndez y Pelayo, 19, 46010 Valencia, Spain; olga.navarro@uv.es; 2 Faculty of Medicine and Health Sciences, Catholic University of Valencia San Vicente Mártir, C/Espartero 7, 46007 Valencia, Spain; david.fernandez@ucv.es (D.F.-G.); olga.forero@ucv.es (O.F.-R.); 3 Nursing Department, Faculty of Nursing and Podiatry, Universitat de València, Menéndez y Pelayo, 19, 46010 Valencia, Spain

**Keywords:** DeepFakes, artificial intelligence, nursing, ethics, technology, education

## Abstract

Background: “DeepFakes” are synthetic performances created by AI, using neural networks to exchange faces in images and modify voices. Objective: Due to the novelty and limited literature on its risks/benefits, this paper aims to determine how young nursing students perceive DeepFake technology, its ethical implications, and its potential benefits in nursing. Methods: This qualitative study used thematic content analysis (the Braun and Clarke method) with videos recorded by 50 third-year nursing students, who answered three questions about DeepFake technology. The data were analyzed using ATLAS.ti (version 22), and the project was approved by the Ethics Committee (code UCV/2021–2022/116). Results: Data analysis identified 21 descriptive codes, classified into four main themes: advantages, disadvantages, health applications, and ethical dilemmas. Benefits noted by students include use in diagnosis, patient accompaniment, training, and learning. Perceived risks include cyberbullying, loss of identity, and negative psychological impacts from unreal memories. Conclusions: Nursing students see both pros and cons in DeepFake technology and are aware of the ethical dilemmas it poses. They also identified promising healthcare applications that could enhance nurses’ leadership in digital health, stressing the importance of regulation and education to fully leverage its potential.

## 1. Introduction

The term artificial intelligence (AI) is somewhat ambiguous, because although it is a subfield of computer science, it can also be related to data processing and text generation as well as tasks that normally require human intelligence, such as reasoning, problem solving, learning and decision making [1]. AI also has a wide range of applications, both specific and general.

Therefore, the influence of artificial intelligence on human life has attracted the attention of various professionals, including healthcare professionals. However, its use in care delivery represents an important duality: it encompasses challenges and recommendations, but also pros and cons [1,2]. The successful implementation of AI requires a clear understanding of the intended purpose and setting achievable goals, as well as determining the potential ethical challenges arising from its use.

In the healthcare environment, the opportunities that AI creates to improve care by means of the optimization of healthcare services and products are becoming more diverse and refined every day. It enables the analysis of large amounts of medical data to detect altered patterns accurately, or to create personalized plans that are more specific than those developed by human perception alone [1]. It also makes addressing language disparities in medical care by means of interpretation services possible [3]. It even facilitates predictive analytics to detect patient vulnerabilities in conjunction with nursing assessment or health monitoring with wearable devices that help identify the best treatment options [4], simplifying decision making, patient care, service delivery and improved healthcare management, which significantly reduces the burden of documentation activities [5]. Therefore, there is a need to train future nursing professionals in AI so that they can lead and participate in digital initiatives as a complement to nursing care delivery [6]. Despite the controversy that this technology generates among nurses [7], some healthcare professionals see AI as a threat to their jobs [8], as do some undergraduate health science students, who believe AI may negatively affect their careers [9].

This raises some ethical, legal, or social issues that involve ensuring the privacy and data control of patients’ personal information related to the use of AI [10], as well as ensuring that the disadvantages will outweigh the associated advantages [11]. Along these lines, it is worth highlighting the important work carried out by the National Cybersecurity Institute (INCIBE) [12] to strengthen digital trust, increase cybersecurity and resilience, as well as the contribution it makes to the digital market for the secure use of cyberspace in Spain. Since the end of 2020, the healthcare sector has become one of the main targets of cyber attacks, due in part to the high value of the data it manages and in part to the criticality of its services. Therefore, the consequences of cyberattacks have a high impact that can affect the correct provision of healthcare services, with direct implication on the patient’s own health [13], which makes it necessary for future nursing professionals to have an in-depth knowledge of cybersecurity and use it to effectively protect information and systems in the healthcare environment.

This is the case with ‘DeepFakes’, which are characterized as content produced by artificial intelligence that appears to be authentic to the eyes of a human being, through systems known as neural networks [14], i.e., they are videos manipulated to make users believe that a specific person, who may be anonymous or a public figure, makes statements or performs actions that never happened. Furthermore, two categories of DeepFakes can be distinguished: deepface and deepvoice. The first is characterized by the superimposition of one face over another, with the aim of falsifying a person’s facial gestures. The second, or deepvoice, is used to imitate or clone an individual’s original voice from fragments. It allows single words or complete sentences to be put together to create speech [15]. Since their emergence in late 2017, they have become a highly controversial technological advance, both legally and morally. However, various experiences speak of the difficult task of distinguishing between authentic images or videos and DeepFakes, which is a major technological and human challenge [16,17,18], among other things, because they immediately reach millions of people through social networks, giving rise to fake news, hoaxes, and fraud [19,20,21].

Broadly speaking, the main risks involved in the use of DeepFakes are blackmail, intimidation, and ideological conditioning [22], and the technology’s links to domestic violence [23,24] pose a danger to privacy, democracy, and national security [18], as convincing changes to faces through this type of technology have the potential to disrupt security-related applications and communications [25]. There are even testimonies of sexual abuse that has occurred based on images and recordings of sexual content that can be shared instantly and without consent [24,26,27], or those studies that mention the potential for DeepFakes to modify our memory and implant false memories [28]. However, it is also worth noting that the current literature also includes a series of positive aspects derived from the use of DeepFakes in different disciplines. Generative adversarial networks (GANs), or the artificial intelligence algorithms, are techniques with enormous potential in the art, industry, engineering, or health sectors, among other areas [29,30].

Regarding the involvement of DeepFakes in healthcare, there is also some controversy about the potential harms and benefits of using this technology. The main risks are related to the rapid dissemination of fake news that may affect the health of the population [31], as well as the publication of false images in scientific articles that could generate confusion in the academic field [32,33] or the disparity of opinions regarding the use of DeepFake in bereavement care. Some authors have observed that the application of this technology in bereavement may alter memories of the deceased, further increasing the negative impact [34]. On the other hand, DeepFake technology in the field of healthcare presents great potential in the field of virtual reality or entertainment videos [18] with a particularly promising focus on doctor–patient communication [30,35], where the application of facial emotion recognition and the potential use of artificial empathy by means of the use of DeepFakes in patient care can be revolutionized. Such is the case of a study that explains the benefits observed in people with Alzheimer’s disease (who have shown positive reactions to interacting with a younger face they can remember) [36] Similarly, they can promote deeper research within the academic and healthcare community by sharing medical video data in clinical settings while protecting users’ confidential data [35]. Today, an increasing number of studies from different parts of the world link DeepFake technology to making more accurate diagnoses in oncologic pathologies as well as improve imaging and radiodiagnostic techniques [37,38,39,40]. Other authors, such as Yang, consider DeepFakes as tools to improve therapeutic adherence, the healthcare professional–patient relationship, as well as an opportunity for professional training [35]. Some authors even find this technology useful for working on compassion and empathy in future health professionals [35,41].

Therefore, this research analyzes the use of AI from the prism of DeepFakes based on the opinions of nursing students interviewed, and evaluates questions regarding the potential advantages and disadvantages that this technology currently poses.

No previous research has been conducted on students in Spain regarding this topic. Therefore, the main objective of this study is to determine how young nursing students perceive DeepFake technology, whether they consider its use ethical and whether they believe it could be beneficial in the field of nursing and care. We are interested in identifying and learning about the positive and negative aspects of the use of DeepFakes in healthcare. Although artificial intelligence has multiple variants and utilities, we focus only on technology based on DeepFakes in this paper.

## 2. Materials and Methods

This study used a qualitative–descriptive design. The qualitative descriptive method aims to provide a detailed account of the phenomenon under investigation. This method is particularly valuable when trying to gain an in-depth understanding of a specific context, experience, or social phenomenon [42]. A thematic analysis approach was used to carry out the data analysis for this research, involving the transcription of text in videos recorded by students on the Information and Communication Technologies course in the third year of the Degree in Nursing at the Catholic University of Valencia during the 2021–2022 academic year.

When carrying out this research, the students were asked to discuss the use of DeepFakes by being shown the following:

1. A video of an advertising campaign in which the face and voice of a famous person in Spain, now deceased, had been used (including deepface and deepvoice).
2. A news item that talked about the need to regulate DeepFakes because of possible legal risks.
3. A news item about a new Microsoft technology that would use DeepFakes so that relatives of deceased people could talk to them through a chatbot (including deepface and deepvoice).

The materials used for the activity were not specific to the health field but of general interest (TV advertisements, press reports). The full formulation of the classroom exercise and links can be found in Appendix A.

The students did not receive any prior explanation from the teachers or any material other than that mentioned in the classroom exercise, nor did the teachers give their opinion on the DeepFakes to avoid conditioning the participants’ answers.

After consulting these materials, students had to search the internet for information about DeepFakes, identifying the possible advantages or disadvantages of using this technology. In addition, they were asked for their opinion on the possible application or usefulness of this technology in the field of healthcare. Finally, they were asked to reflect on Microsoft’s patent from an ethical or healthcare point of view.

In response, the students gave their opinion on the topic by recording a short video lasting a maximum of one minute and 30 s using Microsoft’s Flipgrid tool for an asynchronous online video discussion forum. This web-based tool enables video discussion threads that are stored sequentially once created. It is a secure tool for classroom work, as each discussion thread can only be accessed by an invitation from the teacher. Flipgrid is very user-friendly, and it is designed to be used in a school environment. However, the teacher of the subject developed an explanatory video tutorial that she gave to the students with instructions on how to utilize it. Flipgrid was selected for this activity, because in addition to allowing for the generation of an individual video, it also allows for viewing the contributions of other students and adding comments to the discussion. This tool has already been used by other authors for reflection in the classroom [43] and to stimulate participation in discussions and practical exercises [44,45].

The number of students on this course was 120. The only prerequisite for participation was to be enrolled in the course. Participation was voluntary and required the signing of an informed consent form. The study was conducted according to the ethical and legal rules of the Declaration of Helsinki and the Good Clinical Practice guidelines of the European Union, which states that “precautions must be taken to safeguard the privacy of the research participant and the confidentiality of his or her personal information”. The data collected were entered by the research team into a database for subsequent analysis. The participants’ anonymity was always respected, even concerning the researchers, as personal data and any other identifying elements were eliminated during the transcription process. The Ethics Committee of the Catholic University of Valencia San Vicente Mártir approved the project, with the code UCV/2021–2022/116. The participants did not receive any reward for their participation.

For the subsequent analysis of the information, the students’ opinions were transcribed verbatim and anonymously in a text document using the Word Online transcription tool, to maintain the anonymity of the students. After the transcription (carried out simultaneously with the audio to remain in contact with the primary data generated in the participant’s discourse), transcription errors in the text were checked against the audio analyzed.

Finally, the text resulting from the students’ opinions was categorized by a thematic analysis, following the method proposed by Braun and Clarke [32], using the qualitative data analysis programme ATLAS.ti (version 22) for classification and analysis. The process of analysis in this study focused on the elaboration of mutually exclusive and collectively exhaustive categories.

To ensure the accuracy of the data, a review of the textual elements and their categorization was conducted by one of the researchers, followed by a review by a second researcher [46,47]. This way, the authors broadened, deepened and reduced the possibility of misunderstandings, thus clarifying the meaning and veracity of the information obtained in the testimonies. This verification was also achieved by obtaining the different perspectives of our research team members. Finally, the resulting report was discussed within the research group, and through reflective thinking and critical reasoning, changes were made until a consensus was reached.

## 3. Results

A total of 120 responses were received, and although all of them were analyzed, saturation was reached after analyzing the first 50 exercises. That is, no new categories that had not previously emerged and were not redundant were obtained [48,49,50]. Up to 50 testimonies extracted from the students’ videos were analyzed, and a total of 88 descriptive codes were identified with the help of the software, which were narrowed down to 21 after careful reading, review and discussion among the research team. The codes were grouped into four main themes, corresponding to the three questions asked in the classroom exercise (advantages and disadvantages, the applications of this technology in the field of health, and finally, thoughts on Microsoft’s chatbot.) and six sub-themes (see Table 1).

In response to question 1 of the proposed activity, the students participating in the study noted both advantages and disadvantages of using DeepFake technology.

### 3.1. Advantages

Regarding the economic and social benefits, it is important to remember that the activity was based on the example of an advertisement made using DeepFake technology.

Some students considered the use of deceased celebrities to sell products beneficial and interesting from an economic and commercial point of view. In fact, they indicate that as for the advantages that we can find in the advertising field, for example, *(I think) it is a good tool, as we can make much more attractive advertisements for the consumer* (Participant 21). Participants point out that this technology can have a positive impact on advertising, favouring product sales, generating greater visibility and trust. *Using Deepfakes in this case; it’s a good idea, as a marketing strategy like this could go viral thanks to celebrities or well-known faces, which means that more people will see it and it will achieve a greater impact (Participant 40).*

Related to social impact, they highlight the leisure and entertainment sector as one of the beneficiaries of the use of this technology, in order to finish films or series, even in cases where the actor has died. As one participant points out, one positive use of this technology may have been in Star Wars. *The actress who played Princess Leia died during filming. So, using this technology, they managed to finish the film* (Participant 24). In this case, some students consider it not only appropriate, but also ethical to use DeepFakes to complete films or advertising when the actor has died. *In this case, as it is about a person who has been dead for years, whom everyone knows, and as it is just an advertisement, it would not have any more importance* (Participant 30).

In this regard, students also point out that all *this [DeepFakes employment] would not be very important if we used it for entertainment or as a joke* (Participant 28), emphasizing the permissibility of its use for humour and leisure.

### 3.2. Disadvantages

The students consider that the use of DeepFake technology also has disadvantages and focus on problems related to information hoaxes, cyberbullying, and legal dangers. They are concerned about the difficulty of determining the veracity of information submitted by means of *the use of this technology as it will be much more difficult to distinguish between what is truthful and what is not* (Participant 3). In addition to this difficulty in verifying its veracity, the students also point out the problem caused by a lack of knowledge, indicating that *a person who does not know much about DeepFakes has no reason to suspect that audio-visual content that looks real has been artificially produced* (Participant 10). Another major problem mentioned by participants is the misuse of information for malicious purposes where *people can manipulate a video with a lot of malice to misinterpret words and that can cause big problems* (Participant 16). In this regard, students refer to *identity theft or impersonation, as even crimes such as identity theft can be committed* (Participant 50). They mentioned *that it is easy to tarnish people’s public image and even perpetrate cyber-attacks, with the resulting social repercussions because anyone could use your face and your voice to say or do whatever they want, or to blackmail you (Participant 20). They state that people who use this technology for these purposes can impersonate you and not only do they use your identity, but you must prove that you are innocent, and this could also be used in robberies or thefts* (Participant 2).

Furthermore, students expressed concern about dissemination of false information by impersonating public figures such as politicians, celebrities, etc.

*The most important is the dissemination of false information through impersonation of public persons, the dissemination of this content on the Internet is frightening, since the speed of dissemination of information on the Internet is so fast that in a very short time you can reach millions of people*.

(Participant 18)

In this same context of disadvantages, they refer to health-related problems such as the dissemination of erroneous, incomplete, and even denialist messages. Students find it worrying that by *using this technology the image of a famous person in the media can be used to show an unrealistic denialist attitude towards COVID, for example* (Participant 49). On the same lines, they consider it difficult to tell the difference between a DeepFake and a real video, which leads people to believe anything they see.

*In the health field, this is very worrying because it can easily confuse people and put their health at serious risk*.

(Participant 18)

The students point out that videos using DeepFakes would lead *many people [may] to believe that solutions or diagnoses for any kind of illness can be given by anyone, thus creating an uninformed society* (Participant 11).

### 3.3. Health Applications

In response to question 2, students mention health applications, focusing primarily on the use of therapies and early diagnosis of diseases, mainly in cancer, using imaging techniques in combination with DeepFake technology. In this sense, the technology used to make DeepFakes *can be used*, according to the participants, *in the field of oncology for distinguishing the different types of cancer in X-rays* (Participant 8). The students point out that *this technology helps to diagnose what kind of therapy is needed without wasting resources and time, as this technique allows for a more accurate diagnosis* (Participant 48). Not only could it help to streamline diagnostics and reduce time and resources, *it could also improve the diagnosis of different diseases such as cancer, training future doctors to detect different types of cancer in CT scans or to differentiate diseases in the MRI or irregularities in X-rays* (Participant 45), highlighting its possible use also in the training of medical professionals in diagnosis. Similarly, participants regard this technology as being useful *for learning more about identification in imaging tests as it is being used to recreate fake brain scans with real patient data to detect possible tumours* (Participant 47).

Continuing with the possible learning utilities mentioned, the students consider that *this technology could also be useful for the training of healthcare staff, in case studies, putting us more in the shoes of what day-to-day life in hospital would be like* (Participant 6).

On the other hand, the participants point out that this technology could have application in the nursing care offered to patients and the population. Students identify the use of DeepFake as *a possible way to help people with Alzheimer’s disease by interacting with young faces that they could remember from other times in their lives* (Participant 46). Along the same lines, and to improve communication, the students mention *that it could be useful to employ strategies such as creating familiar and recognizable avatars to deal with sick children and elderly people with cognitive problems* (Participant 10).

Regarding education and health promotion, the participants indicate that *this technology could also help provide veracity for certain messages that we want to convey simply due to these videos featuring people recognized in this field* (Participant 29). They also consider that *an elderly person will find it much easier and more understandable to watch the steps of a treatment on a video explained by their “doctor or nurse” than to read a prescription* (Participant 2).

### 3.4. Thoughts on Microsoft’s Chatbot

In response to question 3, students find negative and positive aspects related to the use of the Microsoft chatbot from the point of view of grief management and coping with the death of a family member.

The students believe that there may be issues with the use of DeepFakes for grief management. They even suggest that *it can be very damaging psychologically* (Participant 38) because *it could have a negative psychological impact on the person, who becomes accustomed to a situation that is not real* (Participant 34). Participants also consider that *the use of this technology to talk to relatives who have already died would make it difficult to overcome grief, as it would only prolong the refusal to accept the loss* (Participant 20). This prolonged and poorly processed grief could, according to the participants, even create a dependency on it, with the need to see someone virtually when they are not real.

*I don’t think that person can move forward in their grieving stage, it can even create a dependency on it, of having the need to see someone virtually when it’s not the reality, it can even evade your real world because of the need to see someone who has died and you don’t get over the grief*.

(Participant 44)

Students also pointed out some positive applications of this technology in the field of grief management, considering that remembering deceased loved ones could have a therapeutic effect *Personally, I think that remembering loved ones who have passed away could have a therapeutic effect in coping with grief in the face of tragic loss.* (Participant 10). From an emotional point of view, they highlight that *this tool would make it possible to have a last conversation with a loved one and help to say goodbye* (Participant 13).

Finally, there are students who are not sure whether to classify its use to ease grief as a positive or negative aspect. *I think it’s not very natural to talk to people who have died, it might even lengthen some people’s grief, but I think for some people it can have a very positive psychological function because it allows you to communicate with someone you love* (Participant 28).

### 3.5. Recommendations from Participants and Future Expectations

The participants mention that *the use of artificial intelligence, should be closely controlled legally and globally, so that it can be used appropriately* (Participant 34) to mitigate many of the problems mentioned beforehand. They point out that it should be a crime against public information, and *it is very important to raise people’s awareness so that they distrust sources that are not reliable* (Participant 35).

However, despite pointing out this need for regulation, students are optimistic and hopeful about the future use of DeepFakes. *I believe that if this technology were to be used in a reasoned way, it would be a great weapon for many centres, especially where we are now, which is in hospitals and hospital centres* (Participant 25). *Deepfakes are in some ways not bad and will make a lot of things easier in the future, but only if they are used properly.* (Participant 42).

## 4. Discussion

AI has begun to be integrated into today’s society and into many aspects of everyday life, as it can be used in increasingly efficient and complex activities in fields as diverse as healthcare, finance, advertising, meteorology, and transport, among others [51]. However, this rapid spread of AI in our society is what has motivated this study, considering that it is necessary for students to develop critical thinking skills in order to understand the correct use of artificial intelligence technology such as DeepFakes and its impact on health.

What is certain is that the use of artificial intelligence to recreate people realistically is a controversial topic for students, who perceive, from their point of view, both benefits and risks in different social environments, in applicability, and problems derived from its use in the healthcare environment, as well as the need to have a legal framework that regulates the responsible use of DeepFakes.

According to the participants, some of the most relevant repercussions they mentioned about the use of DeepFakes not directly related to nursing care and nursing care, were the economic, commercial and advertising benefits, as well as an important social impact on the leisure and entertainment sector. However, they indicate that this technology may not only be appropriate but also ethical, for example, in order to be able to finish movies or series in which the actor has died. In this regard, Franganillo [51] also states that DeepFake technology has changed the creative industry through the recreation, rejuvenation, or “resuscitation” of some characters at a low cost [51]. However, the students who participated in this study also pointed out the negative spectrum of DeepFake technology use. They are concerned about the impersonation and fraud that can be associated with DeepFakes, especially in the face of high-quality realistic imitations on a low budget, as noted by other researchers [52], or the difficulty in detecting a video or image that has been manipulated with DeepFakes [53] and, indeed, multiple studies argue that it is even difficult for a computer or artificial intelligence to detect this difference [17,31]. Therefore, the development of these new artificial intelligence applications invites us to perform prospective analyses of the consequences that the use of tools for the automatic generation of hyper-realistic and personalized audiovisual content could have in different areas of society [54].

In terms of contextualizing the use of DeepFakes and their applicability in the field of health, students felt that this technology could have potential health benefits but also some negative impacts. Among the main advantages, the students believe that it could be a powerful tool for the early diagnosis of different diseases such as cancer, or in the use of therapies in people with Alzheimer’s disease or cognitive impairment, using the combination of imaging techniques with DeepFake technology. Although there is no scientific evidence on the improvement of cognitive impairment, the use of this technology could be an interesting future line of clinical research. On this same line, the study by Falahkheirkhah et al., [39] obtained positive results in the use of DeepFakes, which indicate that it is especially useful in the diagnosis of complex pathologies such as cancer, and even in the field of radiology and diagnostic imaging [40], even for improvements in postoperative results which are being observed thanks to the incorporation of DeepFakes technology [55]. This has also been mentioned by other authors such as Yang, who consider DeepFakes as tools to improve therapeutic adherence, the healthcare professional–patient relationship, as well as an opportunity for professional training [35].

Regarding the negative side of DeepFake technology, students believe that this technology could influence the spread of fake health news and confuse people. This coincides with what was mentioned by other authors who have pointed out the use of this technology in the dissemination of health hoaxes [32], especially in critical moments such as the COVID-19 pandemic [33]. This technology can even affect scientific dissemination in health, generating false images that can be published in high-impact journals [56,57]. In addition, students underline that the use of this technology may affect the emotional health of bereaved people in the long term by preventing them from successfully coping with their grief. In fact, the authors cited above [34] argue that it may have risks, as it could aggravate emotional pain by creating a temporary illusion of seeing the deceased person in a video and giving the feeling that they are still alive, which could prolong the emotional pain. However, other students expressed different opinions, as this is a particularly controversial topic. They point to the possibility of helping people cope with grief in a less painful way. These results are in line with Kidd et al., who argued that having conversations with deceased people could have benefits that can help the bereaved remember happy times spent together, allowing them to better process their emotions and come to terms with their loss [34].

As these students said, DeepFake technology must be properly regulated to avoid manipulation and misleading messages, as it evolves rapidly, so its regulation, and platform policies, as well as automatic detection, must always ensure the integrity of users. In general, the current legal framework is insufficient [58], especially in the field of health. In this regard, the solution to the detection of DeepFakes cannot be considered only a technical problem, but must also be considered a social, cultural, and ethical problem, and solutions must take a multidisciplinary approach [59]. Therefore, as teachers, we must address the issue of what is and is not correct in more depth in order to improve the responsible use of this technology to educate future generations. It is important to adequately train health science students in the ethical and moral dilemmas they will encounter during their professional careers [60,61,62], including AI and DeepFake technology, in order to avoid risks and misuse [63].

## 5. Conclusions

This study shows students’ views on the use of artificial intelligence, specifically DeepFake technology. They have identified negative aspects such as health hoaxes, fake news, problems related to political or personal credibility, among others. They also considered that this technology could have positive uses when it comes to commercial, advertising, and entertainment areas. In the field of health, they point out that this technology can help to improve diagnosis, apply new therapies, and even help in the learning and training of professionals. However, students insist on the need to regulate the use of DeepFake technology in order to avoid misuse and to be able to take advantage of all its benefits.

It is important to include these topics in the training of future nurses, helping to raise awareness of the dangers of artificial intelligence in all its variants, but also of its many uses.

## Figures and Tables

**Table 1:** Themes, subthemes, and codes.

Themes	Sub-Themes	Codes
Advantages	Social and economic benefits	Economic benefits
		Leisure and entertainment
		Advertising and marketing
Disadvantages	Information hoaxes	Disinformation Fake or fraudulent news/Manipulation of public information Fake video editing Misuse of social networks
	Cyberbullying and other legal dangers	Tarnishing or discrediting someone’s public image Cyber attacks Unlawful profit-making purposes Identity theft Loss of privacy
Health applications	Diagnosis and therapies	Clinical diagnoses Cancer New therapies
	Nursing care	Training and education Influencing change in habits Alzheimer’s and cognitive problems
Thoughts on Microsoft’s chatbot	Negative aspects	Failure to cope with grief
	Positive aspects	Improving grief

## Data Availability

The raw data supporting the conclusions of this article will be made available by the authors on request.

## Public Involvement Statement

No public involvement in any aspect of this research.

## Guidelines and Standards Statement

This manuscript was drafted following the Standards for Reporting Qualitative Research (SRQR) for qualitative research.

## Appendix A

Proposed formulation of the activity

DeepFakes: Public opinion and cybersecurity

An advertisement for a well-known brand of beer has recently been launched which features Lola Flores, one of Spain’s most well-known flamenco artists (who died in 1995), as the main character. In this link (https://youtu.be/BQLTRMYHwvE) you can see the “Making of” and how, thanks to this technology, a person can say or do whatever we want them to on video. This opens a debate that has been a public concern for some time: is everything we see true?

Here are two links on this subject for your review:

European police are concerned about DeepFakes and call for the creation of a technology to detect them online (link).

Microsoft patents a chatbot that will allow you to talk to people who have died (link).

This activity consists of finding out about DeepFakes, and setting out the advantages and disadvantages of this technology.

Could it have any potential in the field of health?

What do you think about Microsoft’s patent?

This activity will be carried out on an individual basis. To answer the questions, we suggest you record a video through the debate created by the teacher on Flipgrid. You will have to enter the code we have provided as a password. Your video cannot be longer than 1 min and 30 s.
